# Correlation of Physician Assistant Prerequisite Coursework and Student Success

**DOI:** 10.1007/s40670-025-02326-7

**Published:** 2025-02-15

**Authors:** Sarah Neguse, Darcy Solanyk, Jennifer L. Hellier, David Holmerud, Scott Massey, Cathy Ruff

**Affiliations:** 1 Department of Physician Assistant Studies, Rocky Vista University, Englewood, CO USA; 2 Massey & Martin LLC, New Wilmington, PA USA

**Keywords:** Physician assistant training, Prerequisite science courses, Admissions, Student outcomes, PANCE

## Abstract

This study investigates the impact of prerequisite science courses on student outcomes in a physician assistant (PA) training program. Given the conflicting literature on the necessity of such coursework and the variation in required courses across PA programs, this study aimed to assess whether prerequisite courses are essential for success. One PA program eliminated prerequisite coursework, allowing analysis of impacts on student outcomes. Data from 2018–2021 Centralized Application Service for Physician Assistants (CASPA) records and student performance metrics were analyzed. Stratification analysis and Pearson correlation coefficients were used to assess the relationship between the number of science credit hours and clinical year variables. Results indicate no consistent correlation between completed prerequisite credit hours and student performance on various assessments. Even applicants with minimal (0–1) credit hours performed similarly to those with extensive (> 15) credit hours. There was no statistically significant correlation between prerequisite courses and Physician Assistant National Certifying Exam (PANCE) scores. Additionally, the study found no significant relationship between the nationally benchmarked Physician Assistant Clinical Knowledge Rating and Assessment Tool (patterned off the PANCE) scores and the number of physiology credit hours, but a notable inverse relationship between genetics credit hours and a nationally benchmarked summative examination (End of Curriculum Exam (ECOE)) performance. The analysis revealed that variations in prerequisite coursework did not consistently correlate with student performance across assessments, casting doubt on the necessity of specific science courses for PA program success. However, limitations including fewer candidates with prerequisite coursework requirements, and variability in assessment tools, may influence the generalizability of these results.

## Introduction

The physician assistant (PA) profession is rapidly growing and ranked second (#2) in Best Health Care Jobs, and fifth (#5) in the 100 Best Jobs by U.S. News and World Report in 2024 [1]. With a 10-year projected job growth of 31.3% [2], the profession has a bright future that makes it attractive to many prospective applicants. However, with the limited number of seats available for training future PAs, the admissions process has become highly competitive [3]. Given such a competitive process, selecting applicants who can successfully complete a rigorous PA training program and go on to pass the Physician Assistant National Certifying Examination (PANCE) is of utmost importance. One area of debate in the PA admissions process is the value of prerequisite coursework in relation to student success in PA training.

One of the most extensively measured metrics of student achievement in PA education is the PANCE. Over the last 14 years, a review of PA education literature yielded mixed results regarding whether prerequisite science courses correlated with better student outcomes, namely the PANCE. In 2010, Higgins and colleagues showed that a student’s prerequisite course grades did not contribute significantly to their performance on the PANCE [4]. However, Ennulat et al. in 2011 found that a student’s prerequisite grade point average (GPA) did correlate with performance on the PANCE [5], while Brown’s team in 2013 found no correlation between student success and science prerequisite GPA [6]. Mixed results continued with Andreeff in 2014 showing higher scores in pathophysiology and biochemistry correlated with higher PANCE scores [7], while Butina and colleagues in 2017 found prerequisite GPA had no meaningful effect on PANCE performance [8]. Recently, an analysis of data compiled from the PA Education Association (PAEA) Prerequisite 2021 Report demonstrated that a diverse set of prerequisites existed across 273 PA programs. Notably, the analysis revealed substantial differences in the prerequisite coursework, in both the type of course and the quantity, underscoring the challenge of discerning which courses, if any, are indispensable for a student’s academic success [9].

The DO and MD literature also have mixed results regarding the value of prerequisite coursework. For example, in 2004, Dr. Donna Dixon with the New York College of Osteopathic Medicine of New York Institute of Technology found no correlation between science and nonscience undergraduate GPAs and Comprehensive Osteopathic Medical Licensing Examination (COMLEX) Level 1 and Level 2 scores when looking at one cohort of 171 students [10]. Similarly, Wong and colleagues noted in 2009 that the number of preadmission elective upper-level science courses was not significantly correlated with overall academic achievement for osteopathic medical students [11]. Yet, in 2011, Dixon found that science undergraduate GPA was correlated with COMLEX-USA Level 1 and Level 2-CE (Cognitive Evaluation) scores when looking at multiple cohorts totaling 737 students [12]. In the allopathic realm, the Icahn School of Medicine at Mount Sinai, in 2013, created a FlexMed program targeted toward attracting students to medical school who are from a wide range of backgrounds with majors in areas such as the humanities and social sciences. Traditional prerequisites are waived for these participants with no significant differences in performance on the United States Medical Licensing Examination (USMLE) Step 1 failures, class rank, or honors grades [13].

The Rocky Vista University (RVU) PA Program, established in 2017, initially took a more traditional approach to its prerequisite coursework requirements by requiring a set of science courses commonly needed for entering medical education. During the application process, the program identified that many promising candidates lacked specific science courses, a minimum number of credit hours, or essential lab components, which prevented them from advancing to the interview stage. Consequently, despite their suitability for the program in other respects, these candidates were unable to progress, potentially depriving the program of otherwise well-rounded candidates aligned with its mission. The program consistently required an overall undergraduate GPA as a measure of academic potential, which was maintained, even while specific science prerequisites were reevaluated. The mission of the PA profession is to improve and expand access to healthcare in response to a shortage of primary care physicians; thus, it is critical to select applicants capable of succeeding in rigorous training programs. The RVU PA program aligns with the profession’s mission by emphasizing a curriculum designed to ensure student success on national exams and in their future roles as providers, irrespective of their incoming prerequisites.

Amidst a myriad of opinions within both the PA and MD/DO communities and the challenge the program faced during the interview selection process, a fundamental question persisted: What is the true impact of prerequisite science courses on student outcomes in PA training programs? In response to this question, the RVU PA Program eliminated prerequisite coursework prior to the admissions cycle for their second cohort. This decision was driven by several factors: the limited data in the existing literature supporting the notion that required prerequisites significantly enhance student success; the diverse requirements across different programs that made it challenging to establish a universal standard; and a lack of evidence directly correlating poor student outcomes with cognitive attributes, such as coursework grades. Finally, by adopting a more comprehensive approach, the program aimed to evaluate candidates based on a broader, more holistic range of criteria. This study aims to evaluate the impact of pre-matriculation undergraduate science coursework on student performance in a physician assistant training program, specifically assessing correlations with standardized exam outcomes.

## Materials and Methods

Pre-matriculation undergraduate science and math (i.e., statistics) coursework were collected from four admissions cycles of the Centralized Application Service for Physician Assistants (CASPA) (2017–2018, 2018–2019, 2019–2020, and 2020–2021) for matriculated applicants (*n* = 135) to a PA training program. Specifically, the number of courses, credit hours, and the type of course (lecture, lab, or recitation) were tabulated. Science course topics included General Biology, Microbiology, General Chemistry, Organic Chemistry, Biochemistry, Anatomy, Physiology, Genetics, and their respective labs and recitations. The single mathematics course that was included in the analysis was Statistics. Only verified courses were included unless the student had withdrawn (denoted by a “W”) or had no credit (“NC”). In-progress coursework at the time of CASPA submission was excluded.

The assessments used in this study are nationally recognized, standardized exams designed to measure PA student competency at various stages of training. These include:

- **Physician Assistant Clinical Knowledge Rating and Assessment Tool (PACKRAT)**: A nationally benchmarked self-assessment exam created by the Physician Assistant Education Association (PAEA, Washington DC, paeaonline.org). It consists of 225 multiple-choice questions designed to evaluate a student’s medical knowledge across the PA curriculum. While not a high-stakes exam, its strong internal consistency and performance correlations with later exams, including the PANCE, support its validity.
- **PAEA End of Rotation Exams (EOREs)**: Standardized, specialty-specific exams developed by PAEA to assess student performance after clinical rotations. Each exam consists of approximately 120 multiple-choice questions mapped to PAEA’s national curriculum blueprint. PAEA conducts routine psychometric analysis to ensure reliability and validity, including item performance reviews and statistical benchmarking.
- **PAEA End of Curriculum Exam (EOCE)**: A summative, 300-question multiple-choice exam developed by PAEA to evaluate a student’s readiness for graduation. This exam undergoes rigorous validation, including item analysis, standard-setting, and psychometric evaluation, making it a reliable tool for assessing medical knowledge before the PANCE.
- **Physician Assistant National Certifying Exam (PANCE)**: A high-stakes, nationally standardized certification exam administered by the National Commission on Certification of Physician Assistants (NCCPA, Johns Creek, Georgia, nccpa.net). The PANCE consists of 300 multiple-choice questions assessing a candidate’s competence in medical knowledge and clinical reasoning. The NCCPA employs well-established psychometric methods, including item response theory (IRT), to ensure the exam’s validity and reliability.

For the *End of Rotation Exam (EORE)* in our first three cohorts, we developed assessments using Exam Master, a widely used question bank aligned with PA competencies. For the last cohort and all subsequent cohorts, we transitioned to PAEA’s EOREs to ensure consistency with nationally benchmarked assessments. These assessment tools are widely used across PA programs, have established reliability and validity metrics, and provide meaningful benchmarks for evaluating student progress and readiness for practice.

The students’ performance on the Physician Assistant Clinical Knowledge Rating and Assessment Tool (PACKRAT), Rosh Review’s or PAEA’s summative written examination (End of Curriculum Exam (EOCE)), end of rotation exams (EORE), and PANCE were tabulated. It is important to note that the first three matriculated cohorts of this study (admissions cycles 2017–2018 through 2019–2020) completed EOREs that were created by the program (“homegrown EOREs”) and that the last matriculated cohort (admissions cycle 2020–2021) took EOREs that were provided through the PAEA database. Thus, these data were properly grouped for correlation and regression analyses. Data were organized in Microsoft Excel (Seattle, Washington) and general descriptive statistics (e.g., means, standard deviations) were used to summarize data.

A stratification analysis was employed to determine relationships between the number of science credit hours (0–1 credit hour to > 15 credit hours) against standardized exam outcomes (PACKRAT I, PACKRAT II, EOCE, and EORE) and PANCE scores. Data were analyzed using a descriptive stratification analysis while Pearson correlation coefficient and regression analyses for statistical significance were performed in SPSS software (version 29, IBM Corporation). Statistical significance was defined with a threshold of *p* < 0.05.

## Results

Over the four admissions cycles, there were 135 matriculated applicants to a PA training program, and their pre-matriculation undergraduate science and math coursework were collected. However, one matriculated student withdrew from the program before all clinical year variables were completed, and another matriculated student was moved to a different training cohort that was not included in this dataset. Thus, these students’ credit hours were removed from data analyses (*n* = 133 final participants’ data used for analysis). In the analysis, “credit hours” refers to lecture course hours. Laboratory and recitation hours were included in the total credit hours analysis.

**Fig. 1:**
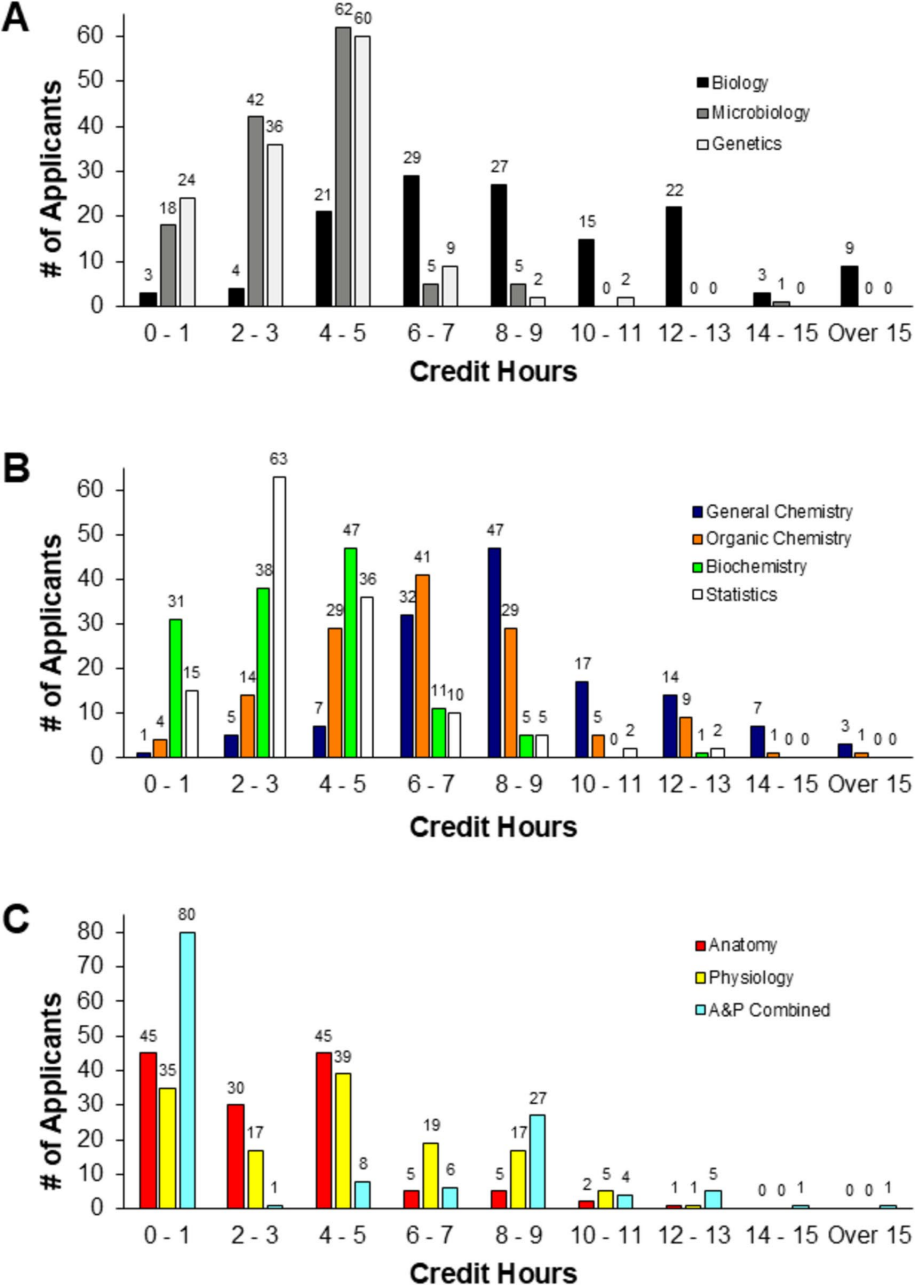
The number of pre-matriculation science and statistics credit hours completed by each applicant prior to matriculating to a PA training program. **A** Biology, Microbiology, and Genetic courses are depicted. **B** General Chemistry, Organic Chemistry, Biochemistry, and Statistics credit hours are shown. **C** Anatomy, Physiology, and Anatomy and Physiology combined coursework are represented. Gaps in data represent that no students completed the number of credit hours for that topic. A&P Combined, Anatomy and Physiology combined course

Overall, there was a broad range in the number of pre-matriculation science and statistics credit hours completed by applicants in each of the different topics (see Fig. 1). Not all students enrolled in or completed some of the pre-matriculation courses. For instance, in general biology, three matriculated students completed 0 credit hours, and nine students completed 15 + credit hours, while the majority completed 4–9 credit hours (*n* = 77, see Fig. 1A and Table 1). These data show the diverse science coursework matriculated applicants finished prior to entering a PA training program.

**Table 1:** Summary statistics of pre-matriculation coursework completed by students (*n* = 133)

*Course topic*	Lecture		Lab		Recitation		Total	
	No. of classes	No. of credits	No. of classes	No. of credits	No. of classes	No. of credits	No. of classes	No. of credits
*General Biology*	319	1145 *x̄* = 8.61 *σ* = 4.12	141	118 *x̄* = 1.40 *σ* = 1.05	9	4 *x̄* = 1.0 *σ* = 2.0	469	1267 *x̄* = 5.81 *σ* = 4.81
*Microbiology*	129	479 *x̄* = 3.60 *σ* = 2.09	57	62 *x̄* = 1.11 *σ* = 0.97	0	0	186	541 *x̄* = 3.16 *σ* = 2.05
*Genetics*	125	444 *x̄* = 3.34 *σ* = 1.99	25	19 *x̄* = 0.83 *σ* = 0.78	7	1 *x̄* = 0.14 *σ* = 0.38	157	464 *x̄* = 3.34 *σ* = 1.88
*General Chemistry*	295	1144.5 *x̄* = 8.61 *σ* = 3.35	188	180 *x̄* = 1.82 *σ* = 1.22	11	1 *x̄* = 0.09 *σ* = 0.30	494	1325.5 *x̄* = 5.48 *σ* = 4.34
*Organic Chemistry*	237	833 *x̄* = 6.26 *σ* = 2.90	153	200 *x̄* = 1.96 *σ* = 1.17	0	0	390	1033 *x̄* = 4.47 *σ* = 3.12
*Biochemistry*	121	423 *x̄* = 3.18 *σ* = 2.23	16	26 *x̄* = 1.73 *σ* = 1.33	0	0	137	449 *x̄* = 3.84 *σ* = 1.73
*Anatomy*	100	401.5 *x̄* = 3.02 *σ* = 2.67	56	57 *x̄* = 1.08 *σ* = 1.00	16	0	172	458.5 *x̄* = 2.94 *σ* = 2.43
*Physiology*	148	531.8 *x̄* = 4.00 *σ* = 3.09	55	66 *x̄* = 1.38 *σ* = 0.84	0	0	203	597.8 *x̄* = 4.09 *σ* = 2.71
*Anatomy/Physiology*	123	436 *x̄* = 3.28 *σ* = 4.25	34	35 *x̄* = 2.19 *σ* = 1.94	0	0	157	471 *x̄* = 5.81 *σ* = 3.88
*Statistics*	143	486 *x̄* = 3.65 *σ* = 2.20	0	0	3	3 *x̄* = 1 *σ* = 1.73	146	489 *x̄* = 4.01 *σ* = 1.96

*x̄* mean, *σ* standard deviation

**Fig. 2:**
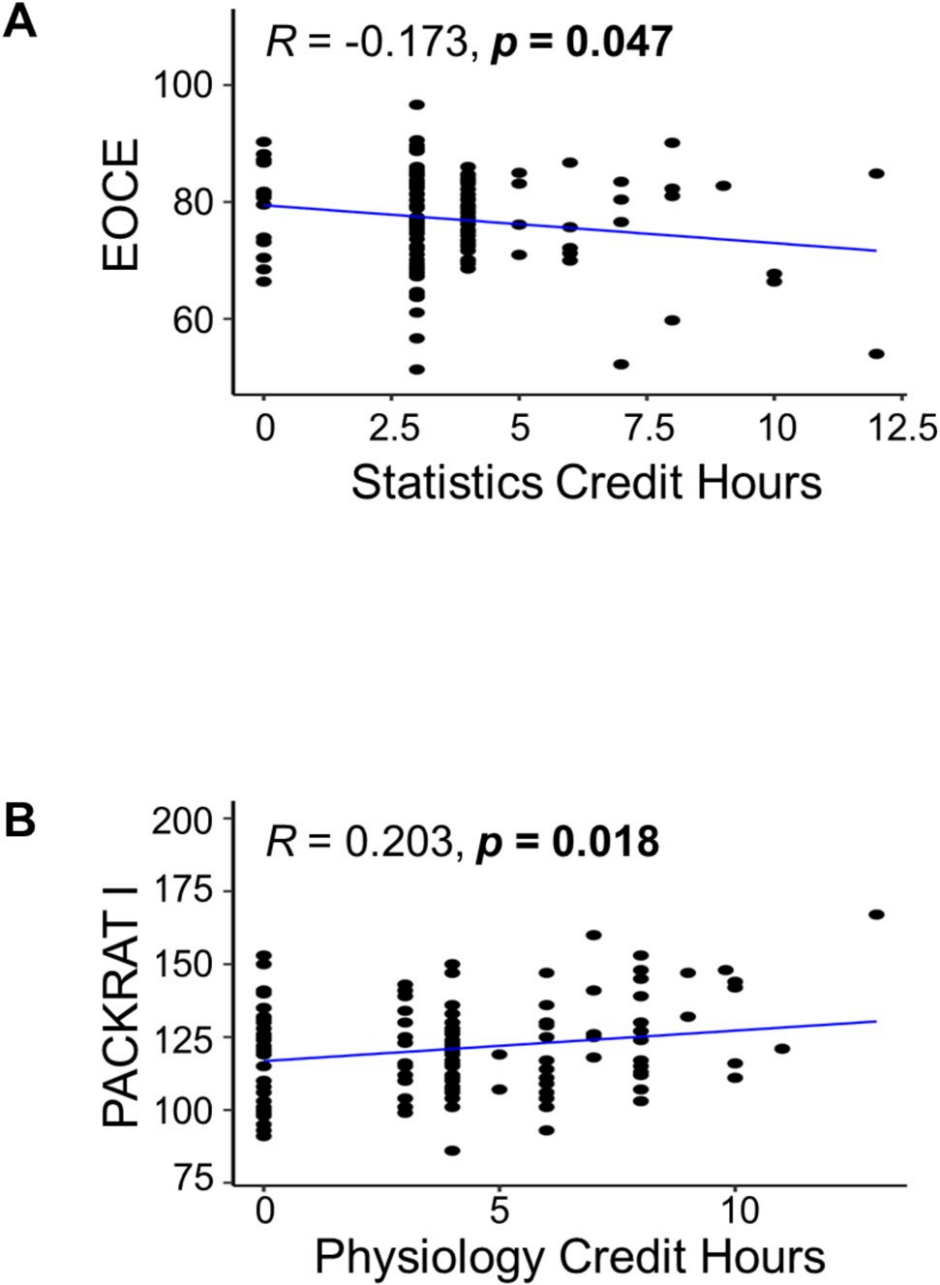
Significant Pearson correlations of student pre-matriculation course credit hours with end of curriculum exam (EOCE = summative written examination (ROSH or PAEA)) scores and PACKRAT I scores (*n* = 133). **A** Scatter plot showing a significant negative correlation exists between the number of credit hours for Statistics and individual EOCE scores. **B** Physiology credit hours vs. PACKRAT I score scatter plot. A positive correlation was observed with the number of completed Physiology credit hours. Significance was determined when *p* < 0.05 (shown in bold)

**Fig. 3:**
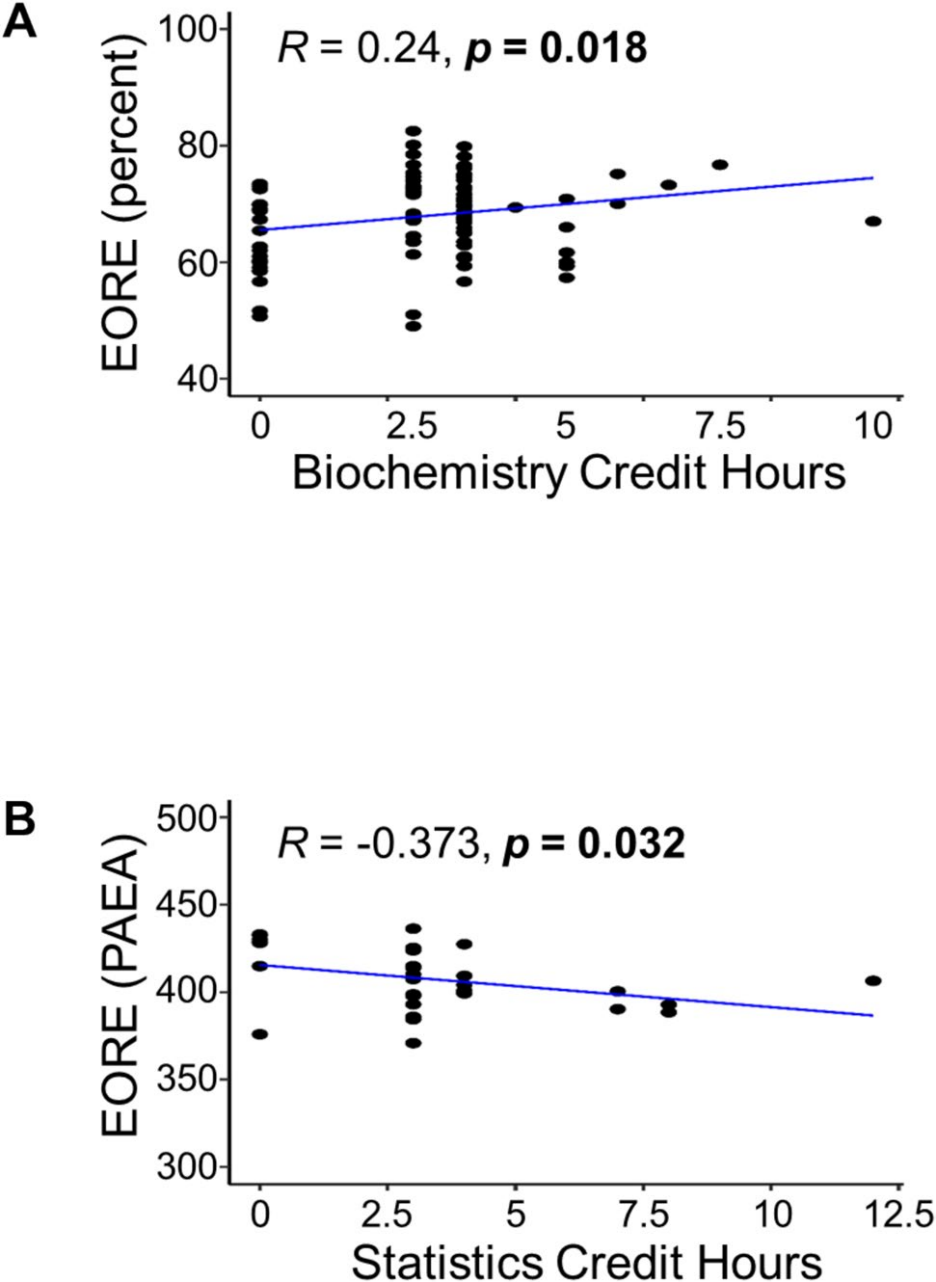
Significant Pearson correlations of student pre-matriculation course credit hours with EORE scores. **A** A scatter graph depicts a significant positive correlation with the number of Biochemistry credit hours completed and an individual’s score on the EORE. Aggregate admissions cycles 2017–2018 through 2019–2020,* n* = 100. **B** Scatter plot showing a significant negative correlation between the total number of completed Statistics credit hours and students’ scores on the PAEA EORE. A single admissions cycle 2020–2021, *n* = 33. Significance was determined when *p* < 0.05 (shown in bold)

**Fig. 4:**
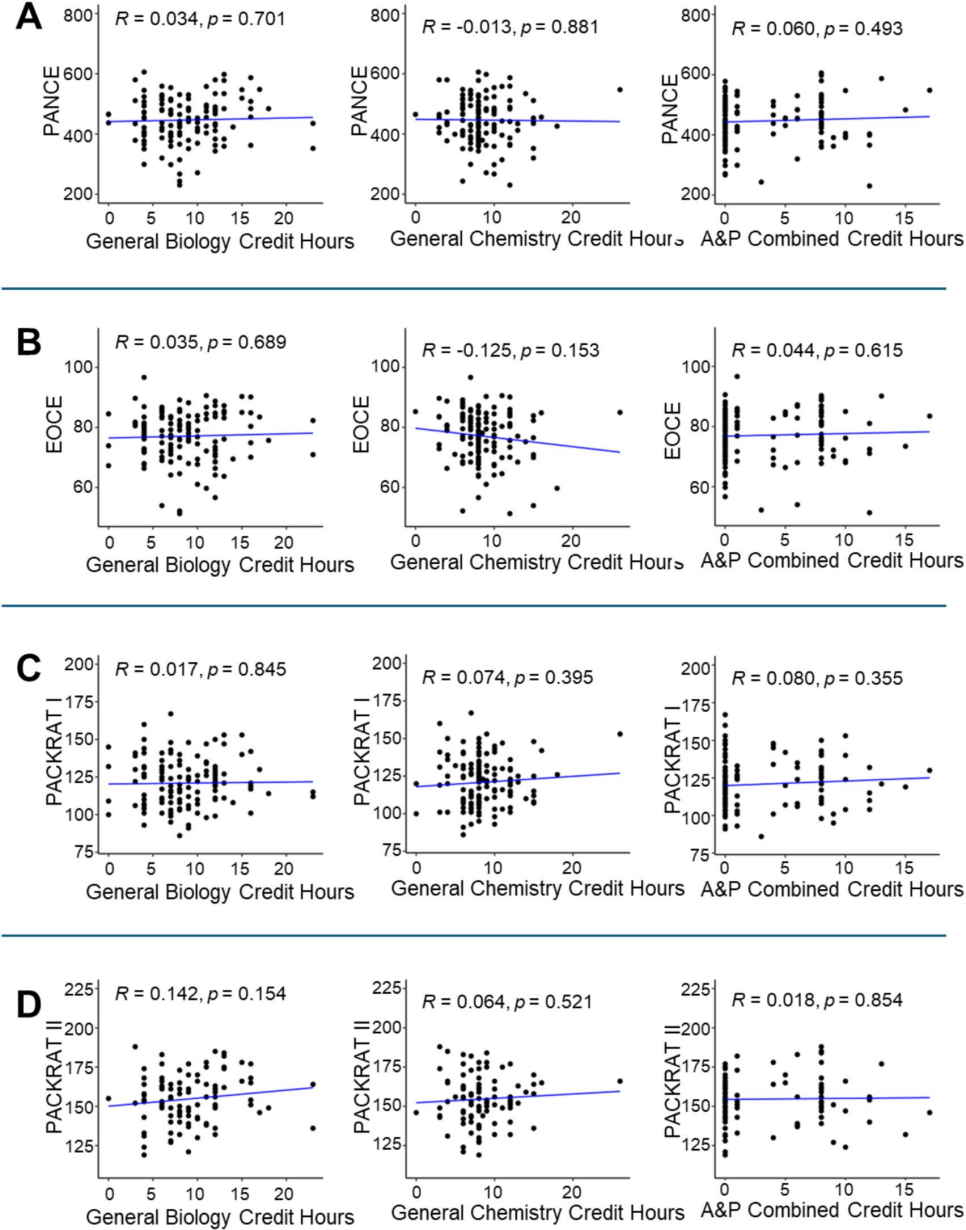
No significant Pearson correlation of student pre-matriculation course credit hours with PANCE, EOCE, PACKRAT I, and PACKRAT II scores (*n* = 133). Most of these correlations were not significant (*n* = 56 of 60; *p*-values ranged from 0.050 to 0.989) and are not shown. Three representative courses (General Biology, General Chemistry, and Anatomy and Physiology combined courses) were chosen to show this lack of correlation. **A** Scatter plots showing the number of credit hours compared to individual PANCE scores. **B** Scatter plots showing the number of credit hours compared to individual EOCE scores. **C** Scatter plots showing the number of credit hours compared to individual PACKRAT I scores. **D** Scatter plots showing the number of credit hours compared to individual PACKRAT II scores. Significance was determined when *p* < 0.05. A&P Combined, Anatomy and Physiology combined course

The authors compared the number of credit hours to each student’s standardized exam outcomes using correlation and multiple regression analyses. There was no consistent relationship between the number of completed prerequisite credit hours and a student’s subsequent performance on the PANCE, summative written examination, EORE, and PACKRAT (see Figs. 2, 3, 4). A majority of these correlations were not significant (*n* = 56 of 60; *p*-values ranged from 0.050 to 0.989, most not shown in figures), indicating that students who completed > 15 credit hours in a science subject did not tend to have higher scores on standardized assessments compared to students who completed fewer pre-matriculation credit hours.

### Credit Hour Comparisons (Lecture Hours Only)

Just four specific correlations were significant compared to the number of credit hours completed. First, there was a significant negative correlation between the number of credit hours for Statistics and a person’s EOCE scores (*R* = − 0.173, *p* = 0.047; Fig. 2A). Second, there was a significant positive correlation between the number of Physiology credit hours and PACKRAT I scores (*R* = 0.203, *p* = 0.018; Fig. 2B). Third, matriculants who took the “homegrown EOREs” had a positive and significant correlation between the number of Biochemistry credit hours and their EORE score (*R* = 0.24, *p* = 0.018,* n* = 100; see Fig. 3A). Lastly, matriculants who took the PAEA EOREs had a negative and significant correlation between the number of Statistics credit hours and their EORE score (*R* = − 0.373, *p* = 0.032, *n* = 33; see Fig. 3B). These data imply that the more lecture hours a matriculated student completed in Statistics before entering PA school, the lower the EOCE and PAEA EORE scores that resulted were. Whereas the more lecture hours a matriculated student completed in Physiology and Biochemistry before PA school, the higher their PACKRAT I and homegrown EOREs scores were.

### Total Credit Hour Comparisons (Lecture, Laboratory, and Recitation Hours Aggregated)

**Fig. 5:**
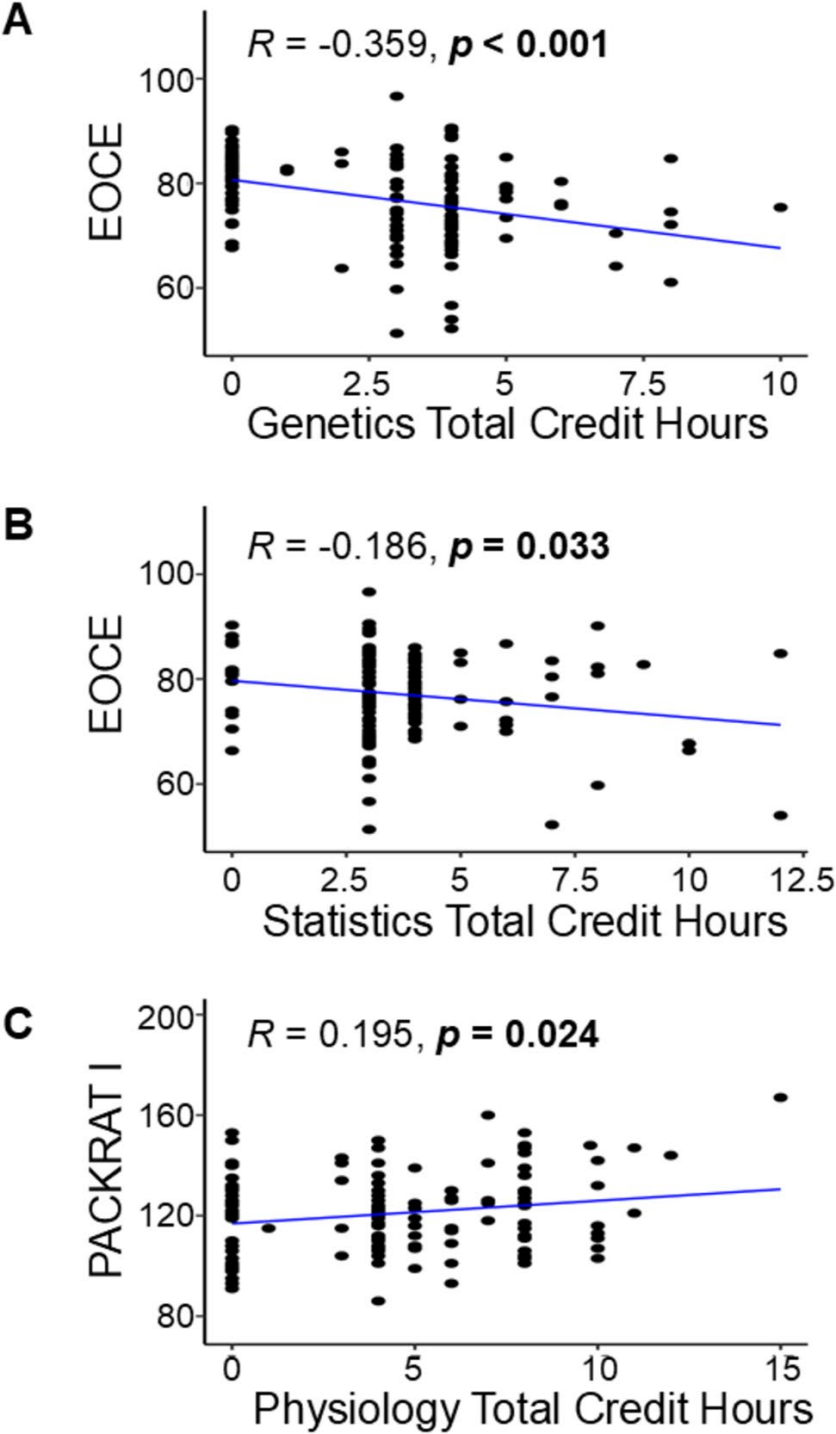
Significant Pearson correlations of student total pre-matriculation course credit hours (including laboratory and recitations) with EOCE or PACKRAT I scores (*n* = 133). **A** Scatter plot showing a significant negative correlation between the total number of Genetics credit hours and students’ scores on EOCE. **B** A scatter graph depicts a significant negative correlation with the total credit hours in Statistics and an individual’s score on the EOCE. **C** The total number of Physiology credit hours is significant and positive correlation with a student’s PACKRAT I score is shown. Significance was determined when *p* < 0.05 (shown in bold)

We wanted to determine if including the number of laboratory and recitation hours with the lecture credit hours would influence matriculants’ scores on standardized exam outcomes. We found three courses that had significant correlations. Both Genetics (*R* = − 0.359, *p* < 0.001) and Statistics (*R* = − 0.186, *p* = 0.033) were significantly and negatively correlated with EOCE (see Fig. 5A and B, respectively). Conversely, Physiology coursework (lecture and laboratory hours combined) was positively correlated with a student’s PACKRAT I score (*R* = 0.195, *p* = 0.024; see Fig. 5C). These data suggest that Physiology coursework may be beneficial early in a student’s training, particularly for the PACKRAT I assessment, which is administered soon after the didactic year concludes.

## Discussion

These findings highlight that the number of science credit hours completed by students entering a PA program may not significantly influence their performance on nationally benchmarked assessments. This supports our initial hypothesis that student success in PA programs is not necessarily contingent upon completing specific prerequisite courses. Our results align with the findings of both Higgins and colleagues (2010) and Butina and colleagues (2017). However, it is important to acknowledge the limitations of our study, including data obtained from a single PA program, fewer matriculated students with prerequisite coursework requirements, and reliance on two different summative exams for assessment. Additionally, there is considerable variability in uncontrollable external factors such as course content (Biology learning objectives between institutions, lower vs. upper division coursework, etc.), institution type, and the nature of the coursework undertaken. Future studies could include a larger sample size across various PA programs, allowing for a more nuanced understanding of how prerequisite coursework influences outcomes.

It is noteworthy that the only consistent prerequisite for the PA program was the applicant’s overall undergraduate GPA. This criterion was maintained as a measure of the applicant’s general academic abilities and potential for success in rigorous graduate-level coursework and was considered for all four cohorts included in this study. The focus on overall GPA rather than specific science courses may have allowed the program to admit a diverse range of students with varied academic backgrounds while still ensuring a high standard of academic competence. This holistic approach to admissions, in which applicants are evaluated on a wide range of criteria, rather than solely academic performance, may explain the lack of correlation between specific science prerequisites and student outcomes. The reliance on overall GPA rather than specific course completion aligns with the broader findings of this study, suggesting that general academic performance is a more reliable predictor of success in PA training than the completion of science prerequisites.

By elucidating the impact (or lack thereof) of prerequisite coursework on student performance, our study contributes to the ongoing dialogue surrounding admissions requirements and pre-matriculation preparation in PA education. These findings suggest that PA programs may benefit from reassessing the weight placed on prerequisite coursework in favor of a broader evaluation of applicant potential, which could include non-cognitive attributes. A comprehensive understanding of the relationship between prerequisite coursework and student outcomes could inform data-driven decisions regarding admissions criteria, curriculum development, and pre-matriculation support programs. This understanding is crucial as it challenges long-standing assumptions about the necessity of specific prerequisite coursework, potentially transforming how PA programs approach student admissions and preparation. Data from more students across multiple PA programs should be gathered and analyzed to determine if the results of this study are relevant to the broader PA education community. If these results are indicative of a broader trend wherein prerequisite coursework has minimal impact on student outcomes, PA programs may opt to reassess their admissions criteria and focus resources on other areas of student support and development. Additionally, alternative predictors of student success might be evaluated. This would enable a shift from emphasizing cognitive attributes like GPA and prerequisite coursework to examining non-cognitive qualities such as self-discipline, perseverance, and motivation, which could potentially play a more significant role in admissions decisions. Although outside the scope of this current study, this concept would be an important and relevant area of future study.

Our findings may also be relevant to educators and policymakers in other health professions education programs, prompting a reassessment of prerequisite coursework requirements and fostering collaboration in developing holistic admissions processes. On a societal level, optimizing admissions criteria based on evidence can lead to more efficient educational pathways, potentially reducing the time and financial burden on students and ensuring a more diverse and well-prepared healthcare workforce. This study underscores the importance of evidence-based decision-making in PA program admissions processes and highlights the need for continued research to inform best practices in admissions and curriculum design. We are eager to expand this research to gain a deeper understanding of these relationships by involving other PA programs. This will enable a larger analysis and may limit generalization, providing a more balanced perspective.

## Conclusion

This study provides valuable insights into the impact of pre-matriculation science coursework on the academic performance of students in a PA training program. The findings suggest that the number of completed science credit hours prior to matriculation does not consistently correlate with success on standardized assessments such as the PANCE, PACKRAT, EORE, and EOCE. While some specific courses like Physiology and Biochemistry showed a positive correlation with certain exam scores, the overall trend indicates that traditional science prerequisites may not be as critical to student success as previously assumed.

These results support the notion that PA programs could benefit from reevaluating the emphasis placed on prerequisite coursework in their admissions processes. Instead of focusing solely on specific science courses, a more holistic approach that considers an applicant’s overall academic performance, as indicated by GPA, along with non-cognitive attributes such as perseverance, motivation, and adaptability, may better predict success in PA education.

To further validate these findings, future research should include larger sample sizes across multiple PA programs to determine achievement in PA programs.

By expanding this research and involving a more diverse range of programs and students, we can explore if the lack of correlation between prerequisite coursework and student outcomes observed in this study is consistent across the broader PA education community. Additionally, exploring alternative predictors of student success, including non-cognitive factors, could lead to a more nuanced understanding of what contributes to academic and professional success and can help programs develop more informed, evidence-based admissions criteria. This could ultimately lead to more effective and equitable selection processes, better-prepared students, and a more diverse and capable healthcare workforce.

## Data Availability

The data that support the findings of this study are not publicly available due to institutional privacy policies and confidentiality agreements. Access may be granted upon reasonable request to the corresponding author, subject to institutional review.

## Declarations

RVU IRB Approval #: 2023–032-Exempt.
